# Chicago Pathological Society—Regular Meeting, August 13

**Published:** 1888-09

**Authors:** W. Whitford


### CHICAGO PATHOLOGICAL SOCIETY.

Reported by W. Whitford, M. D.

Stated meeting, August 13; President I. N. Danforth, M. D., in the Chair.

The session opened with the report by Professor G. F. Lydston, of a case of "Diffuse Syphiloma of the Tongue (with exhibition of the patient), and Excision by Galvano-cautery."

The patient, a man of 29 years of age, contracted syphilis at the age of 18; a hard chancre, which inflamed and caused paraphimosis, which lasted about a month and left the mucous membrane of the penis in an irritable condition, with frequent appearance of fissures and ulcerations. Secondary symptoms appeared about four months after the primary sore, and a pronounced papulo-pustular eruption appeared and resulted in considerable scarring of the skin. After the secondary period became manifest the physician in charge of the patient put him upon mercury for about three months, when the patient went to the Hot Springs of Arkansas. He remained at the Springs three months. He used a large amount of mercury by inunction, and was badly salivated twice. He then returned home and remained two months, after which he again went to the Springs and remained for eleven weeks, during which time he took mercury internally quite freely. He again went home and was apparently well for three months, when mucous patches and eruptions broke out, and sores appeared upon the penis. About five years after contracting syphilis the patient was under the care of a physician who also prescribed free mercurial inunction, and that treatment was continued for about six months. He improved for a time and then quit treatment, but again got worse and went back to the same physician, and for at least eighteen months again took mercury in considerable quantity. He then went to Mount Clemens, Michigan, and remained about fifteen months.

He consulted Professor Lydston in 1884 for the first time. He had then unequivocal cerebral syphilis, and tuberculous squamous syphilides in considerable number were scattered about the forehead, forearms, and thighs, with a few scattered squamæ upon the trunk. Scars of former pustular syphilides were visible upon the forehead, forearms, and legs; the tibæ and sternum were excessively tender, and considerable pain was experienced at night. Cephalalgia was a constant symptom and gave the patient intense suffering. Gummy ulceration, with several fissures, existed upon the penis just back of the corona. The tongue was rough, and coated with thick, dirty, yellowish gray fur, with marked dryness in the centre and posterior part of the organ. Several tubercular nodules were also observable, one in the centre of the tongue and the others near its base, those upon the right side being especially marked.

The treatment at this time was cod-liver oil and iron, these being ordered on account of the patient's marked debility. The mixed treatment and the iodide of potassium in saturated solution were given, the iodide being run rapidly up to a dose of 300 grains per diem, and maintained at that point for three weeks, the disease being very resisting to treatment until the end of that time. Local applications of the acid nitrate of mercury were made to the tongue from time to time, and with apparent benefit. As soon as the symptoms indicated that the disease was yielding to treatment the amount of iodide of potassium was diminished to about sixty grains a day, the tonics being meanwhile continued.

The case was pronounced cancerous by the attending physician and several others who saw it in consultation.

Upon examination, the tongue was found filling the mouth and pressing upon the teeth in such manner that it had become eroded and ulcerated. Salivation was profuse; a deep ulcer existed at the centre and back part of the organ; the enlargement seemed to be rather more marked upon the right side. The submaxillary glands were slightly enlarged. There were no other lesions present upon any part of the body. As he experienced great difficulty in masticating and swallowing, he was subsisting entirely upon fluid food, and never having been very robust he had become greatly emaciated.

The treatment instituted was chiefly tonic, in conjunction with an alterative mixture containing iodide of sodium and the chloride of gold; milk punches were allowed, and he was instructed to take as much sweet cream and milk as he could without creating digestive disturbance. Local applications were made of a compound of carbolic acid, iodine, and menthol in mild strength. There was apparent improvement at first, but the tongue began to enlarge and the case presented a more serious aspect.

Mild inunctions of mercury were tried, but they seemed to aggravate the case.

Punctate cauterization with the galvano-cautery was tried with some reduction in the size of the tongue. Soon thereafter sloughing began and destroyed the greater portion of the right side of the organ, the remainder of it being undermined and eroded, and the floor of the mouth extensively invaded by the central ulceration, of which mention has already been made. He grew worse, regardless of whatever treatment was resorted to, and as there was no prospect of saving any of the tongue, and with a view of getting the foul mass out of the way, excision of the organ, with the galvano-cautery wire, was performed.

If it should be questioned whether or not he was justified in excising the tongue if he believed it to be syphilitic, he stated that the remnant of the anterior half of the organ was attached by so narrow a pedicle from before backwards that, if healing had been possible, the tongue would have been practically useless. The base of the tongue was a mass of hyperplastic ulcerating tissue, and the degenerative process was apt to extend to the pharynx and larynx.

Dr. E. P. Murdock reported "A Case of Superfœtation," and stated that in the winter of 1887, he was consulted by Mrs. C——, aged 26 years, suffering with chronic endometritis, and with a history of a miscarriage some three months before she consulted him. She gradually improved under treatment until the spring of 1886, when he was called to see her for persistent vomiting due to pregnancy. She had a great dread of a miscarriage, and used every effort to avoid it; but in the month of July, 1886, the third month of gestation, the fœtus was suddenly expelled one night after a few severe pains. She made a good recovery, and again returned for treatment with a view of getting entire relief from her old trouble.

In February, 1887, she again presented symptoms of pregnancy, and about the first of March she called Dr. Rittenhouse to relieve her from pain and flooding, which he did very effectually, and assured her that the fœtus had not been expelled. About four weeks later Dr. Rittenhouse again saw her suffering with the same symptoms, and assured her that she was still pregnant, and that no miscarriage had occurred. Dr. Murdock saw her in May and was informed of the treatment she had had, but he could not agree in the prognosis that had previously been made in her case.

He found the uterus considerably enlarged and tender, but not soft and elastic, and not so large as it should be in the fourth month of pregnancy. He advised local treatment, but his advice was not followed, and he did not see her again until September. She was then expecting her confinement in December, and had all the symptoms of a fairly progressing pregnancy. He then gave it as his opinion that she had had a miscarriage in March or April, and that a succeeding pregnancy was in progress, and told her that her confinement would not occur before April 1.

Months passed on and labor did not occur, but it was difficult to understand how a miscarriage at the third month could take place and no fœtus or placenta be found. The month of April passed and still labor had not occurred. During these months Dr. Murdock saw her frequently, on account of severe pain in the uterus, diarrhœa, and occasional attacks of fever. May 2, 1888, thirteen months after the beginning of pregnancy, he was called to attend her in labor, which progressed and terminated normally until delivery of the placenta, which presented such abnormal characteristics that he examined it carefully and found imbedded in its margin a fœtus of about three months' development, and to it attached a placenta completely flattened and mummified, which explained the matter, and induced him to present the specimen with a report of the case to the society.

The history seems so clear, and the entire account of the symptoms and conditions so clearly remembered and stated by the patient as to leave little doubt of a condition which, he said, for want of another term he called *superfœtation*, although it is not such a condition as has been heretofore reported under that name, if such a condition as superfœtation ever exists.

The literature on the subject is quite limited, with the writers divided in opinion on the subject as to whether such a condition exists or not, those who deny the existence of superfœtation basing their arguments upon scientific reasons based upon the changed condition of the organs, to-wit:

1. The changed physiological action of the organs.

2. The firm mucous plug which closes the mouth of the uterus.

3. The changed condition of the decidua preventing communication with the tubes—that is, the general suspension of the functions of conception which exists during the period of gestation, and often extending entirely through the period of lactation.

In the case reported the objections do not exist. First, gestation had undoubtedly ceased, though the fœtus remained in utero. The mucous plug had evidently been displaced by the occasional hæmorrhage, and in fact there are strong reasons to believe that all the processes of ovulation and menstruation had returned after the death of the fœtus.

Dr. Goodell, of Philadelphia, reports a case in which a dead fœtus remained in utero fifteen years, during which time menstruation was regular and profuse, and he finally removed the fœtus, or rather the fragments of bones, from the uterus, and his patient died of sepsis after the operation. This appears to have been a fœtus of seven or eight months' development, while in the case now reported it could not have been more than ten weeks.

Those who hold to the possibility of superfœtation depend entirely upon reported cases for proof. In many of these cases there might have been a double uterus, and the functions of each acting separately, as is undoubtedly the case in some of the lower animals, the rabbit, French coney, etc. Indeed, Prof. Fordyce Barker reports such a case in 1885 coming under his own observation. In this case one child was born nine months after marriage, and seventy-four days later another child was born; both lived and prospered. Eismann, Burdack, Des Granges, Fornier, Velpeau, E. A. Purdy, of New York, Dr. Amos Sawyer, of Hillsboro, Illinois, and a few others have reported cases of duplex gestation with a variance from 70 to 168 days in delivery. In one of these cases Blumenbach accounts for the phenomena on the theory of a double uterus, but a post-mortem upon the woman four years after the birth of the children, when death came from some other cause, the pelvic organs were found normal. With these facts before us we are almost compelled to admit the possibility of superfœtation in rare instances. But it is important to make a distinction between superfœtation and superfecundation—that is, superfœtation being the conception and gestation taking place in a uterus where a fœtus already exists, perhaps two or three, or even four or five months, while superfecundation is the impregnation of two ova within a few days and before any physiological changes could take place in the female organs of generation. Thus, the case reported by Howard, where a woman gave birth to a white child and a black one at the same confinement, could not be called superfœtation, but superfecundation; so with the case of Mr. Adam Vettle, of Nokomis, Illinois, who bred a mare to a stallion, and five days later to an ass, and she brought forth a horse colt and a mule.

Many cases of superfecundation have been reported, and there can be offered no physiological argument against them; but it is quite different in superfœtation. So far as he knew, the case which he had reported stands alone, as he was not aware of any other case of the kind on record. He regarded it as a case of considerable interest, though by no means so wonderful as the cases of two living children being born from the same uterus at an interval of 168 days.

Dr. L. L. Gregory read a paper on "Primary Cancer of the Kidney," reporting a case, and exhibited the diseased organ. After reviewing the literature on primary cancer of the kidney in a general way, he said: I shall now give the history of a case coming under my own observation a few weeks since in St. Luke's Hospital, and shall present to the society for inspection the tumor removed, a portion of the vena cava distended to six times its normal size, with an organized blood clot, the heart, and also the kidney of the opposite side.

The patient was of Swedish parentage, 27 years of age, unmarried, and a seamstress by occupation. The family history as regards disease is very good. The brothers and sisters are all healthy. The patient's health previous to this illness was uniformly good. Her entrance into womanhood was not marked by any trouble, and her menses have been regular and normal. She entered the hospital the 11th of April last, weak, emaciated, and of a sallow complexion. Last November she first noticed a small lump in her abdomen a little above and to the right of the umbilicus. At first there was no pain, but about Christmas she began to suffer pain at intervals in the region of the right kidney. The tumor, on examination when she entered the hospital, was oblong and nodular in shape and about 3¼ inches in its shortest diameter. One month from this time the tumor, which had steadily increased in size, was removed. It extended from a line 2½ inches to the left of the umbilicus to the right side of the abdominal wall, and from the ninth rib above to the line a little below the upper border of the pelvis. Posteriorly it occupied the whole of the lumbar region on the right side. On percussion it was dull, diminishing in flatness from the right to the left side. The tumor was slightly movable from the right to the left side. The margin between the growth and the liver was with difficulty made out, owing to the adhesions between them.

The week previous to the operation the urine was examined with the following result: Sp. gr. 1.018; alkaline in reaction; no albumen; no sugar; no blood. A few triple phosphatic crystals; mucus in large amount; squamous and round epithelial cells, and a few pus-cells.

The tumor was also aspirated and about two ounces of fluid drawn off, which was found to contain albumen, 25 per centum by volume; blood globules, large cylindrical casts composed of round nucleated cells; occasional large, round and polygonal cells, nucleated, isolated and in patches; broken-down connective-tissue shreds, and much granular matter; also groups or nests of large nucleated granular cells.

The patient gave a history of chronic constipation, but an attack of diarrhœa delayed the operation for more than two weeks. The liver, spleen, heart, and lungs were normal. The right kidney was somewhat hypertrophied. The stomach was pushed to the left side, and she was unable to take but little food at a time without distressing her. The last ten days before the operation she suffered constant pain in the tumor and darting pains in the lower extremities.

